# Self-interest overrides rank-reversal aversion in resource distribution

**DOI:** 10.1038/s41598-024-70225-9

**Published:** 2024-08-24

**Authors:** Minyoung Kim, Kun Il Kim, Hackjin Kim

**Affiliations:** School of Psychology, Korea University, 145 Anam-ro, Seongbuk-gu, Seoul, 02841 Republic of Korea

**Keywords:** Distributive justice, Fairness, Self-interest, Equality, Moral strategy, Psychology, Human behaviour

## Abstract

The equitable allocation of resources has long been a central concern for humanity, prompting extensive research into various motivations that drive the pursuit of distributive justice. In contrast to one of the most fundamental motives, inequality aversion, a conflicting motive has been proposed: rank-reversal aversion. However, it remains unclear whether this rank-reversal aversion persists in the presence of self-rank. Here we provide evidence of rank-reversal aversion in the first-party context and explore diverse moral strategies for distribution. In a modified version of the redistribution game involving 55 online-recruited participants, we observed rank-reversal aversion only when one’s rank was maintained. When participants’ self-rank was altered, they tended to base their behavior on their new ranks. This behavioral tendency varied among individuals, revealing three distinct moral strategies, all incorporating considerations of rank-reversal. Our findings suggest that rank-reversal aversion can indeed influence one’s distribution behavior, although the extent of its impact may vary among individuals, especially when self-rank is a factor. These insights can be extended to political and economic domains, contributing to a deeper understanding of the underlying mechanisms of distributive justice.

## Introduction

Equality has been a longstanding principle in the allocation of resources^1^. Numerous behavioral and neural studies have indicated a preference for reducing inequality as a fundamental principle of distributive justice^2–5^. For instance, individuals have been observed to penalize those violating equality norms, even at a cost to the punisher^6,7^.

However, a counteracting inclination toward inequality aversion lies in the desire to maintain stable hierarchies. Social hierarchies are often perceived as comprehensible and preferable, reinforced by both social structures and personal beliefs that support their stability^8–10^. This inclination to prevent rank reversal conflicts with the pursuit of equality, creating a trade-off between inequality aversion and rank-reversal aversion. For example, taxation policies globally attempt to redistribute wealth from the rich to the poor while preserving social order and the interests of all income groups^11^.

Previous studies employed the redistribution game to measure rank-reversal aversion, where participants decided whether to redistribute money to others, with potential offers involving the reversal of relative ranks. The findings indicated a reluctance to overturn existing hierarchies, even at the expense of violating fairness norms, a trend observed across various cultural and sociodemographic groups^12,13^.

Yet, it remains unclear whether this rank-reversal aversion is evident in a first-party context. Previous redistribution game studies focused on selecting payments for anonymous individuals without impacting the decision-maker directly. However, rejecting unfair offers for oneself and for others involves different mechanisms^14^. Also, while people show inequality aversion even when their payoff is not affected, emotional arousal is found only when the unfair offer is self-related^15^. These findings suggest that decision-making may differ when the unequal situation directly affects oneself. Furthermore, even when the unequal treatment is not directly given, simply being involved in such a scenario can have an impact on one's decision. For instance, individuals' wealth status significantly influenced the degree of distributing wealth and protecting equality for others^3^. Hence, introducing self-interest as a factor may affect the outcomes of prior research.

In this study, we expanded on previous research by introducing the redistribution game into a first party context. The experimental design mirrored the original version where participants were instructed to select the final endowments for anonymous individuals. However, participants were also presented with information about their own potential earnings, establishing both absolute and relative ranks among players. The redistribution process did not affect participants’ endowment but involved transferring points between other players. As participants always started as second place, their relative self-rank could change after the redistribution process. To differentiate the rank-reversal of others from the rank-reversal of self, we labeled the former as ‘general rank-reversal’ and the latter as ‘self-centered rank-reversal’. Therefore, the combination of the reversal of others (general rank-reversal: rank-reversal, no rank-reversal condition) and the reversal of self (self-centered rank-reversal: upward, maintain, downward) resulted in a total of 6 conditions.

**Figure 1:**
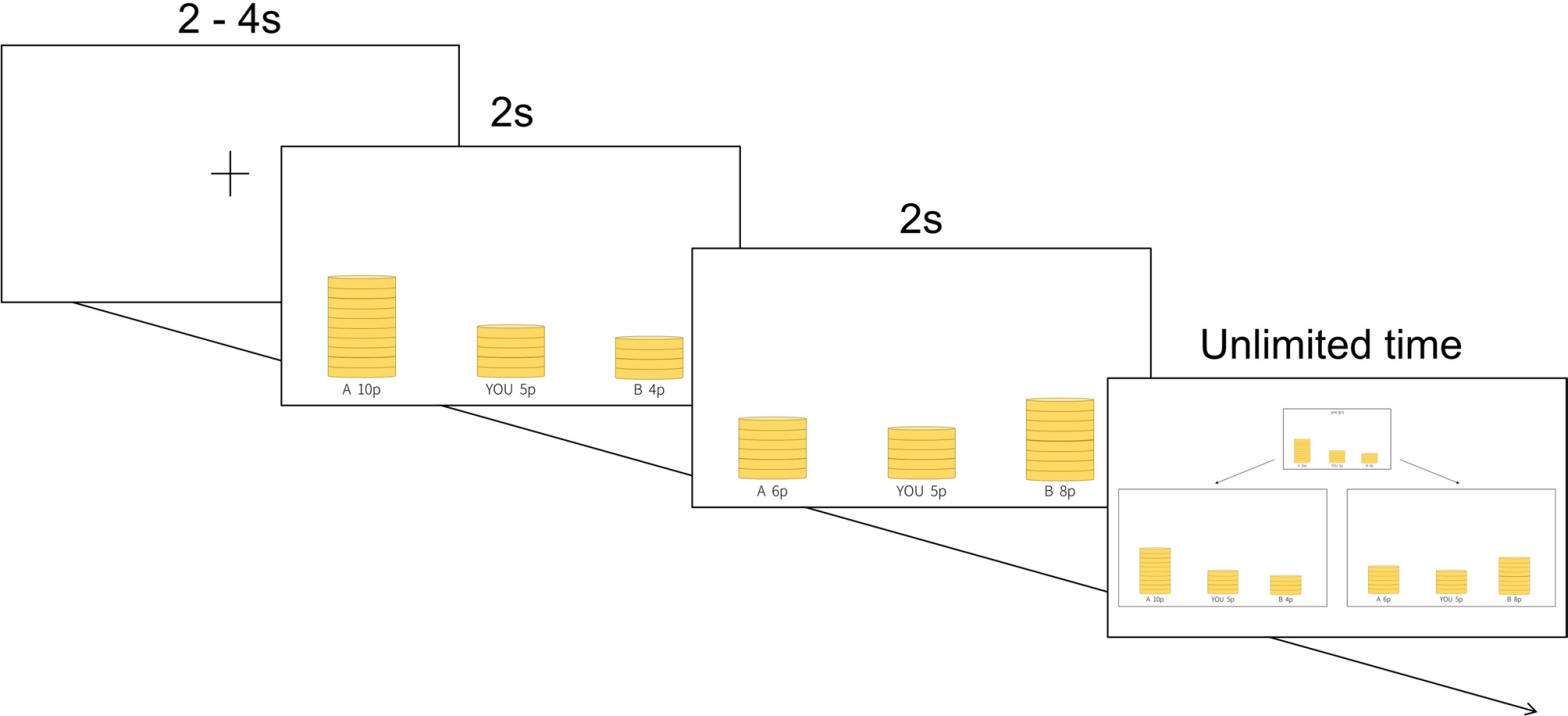
Time sequence of the modified redistribution game. The initial offer and the redistributed offer were displayed for 2 s each, and participants used keyboard to choose whether to reject or accept the redistributed offer.

For example, following their initial offer, participants were presented with the redistributed offer (Fig. 1). Although the quantity of points participants could acquire remained unaffected (e.g., 5 points), their ranking shifted from second to third. Participants might choose to accept the redistributed offer to rectify the inequality between A and B, adhering to the fairness norm. Alternatively, they might reject the offer due to rank-reversal aversion, prioritizing the preservation of a stable hierarchy over equality. They could also reject the offer to avoid ending up in last place, consistent with previous study where individuals in second-to-last place tend to distribute money to those who have more resource than to who have less, due to the fear being overtaken by individuals lower in rank^16^. This aversion to being in last place may lead participants to reject a more equal offer even when the distribution of points remains the same. Various motives, such as aversion to inequality, aversion to rank-reversal, and self-interest (aversion to last place), may be considered in decision-making.

Our research question revolves around how these conditions for self-centered rank-reversal and general rank-reversal impact the decision-making process of distribution. We hypothesized that, firstly, in situations where self-rank is reversed, self-interest would be the most significant motive, with individuals more likely to accept a redistributed offer when their self-rank increases and reject it when their self-rank decreases. Secondly, when the self-rank is maintained, there is an opportunity to consider others, and general rank-reversal aversion may come into play. In addition, individual differences in the motives valued by each person may exist.

## Results

### General rank-reversal aversion expressed only when self-rank is preserved

The task comprised 6 conditions with combinations of two general rank-reversal conditions and three self-centered rank-reversal conditions. Rejection rates were calculated for each condition to distinguish various motivations underlying the decision-making process.

The 2 (General rank-reversal) × 3 (Self-centered rank-reversal) two-way repeated measures ANOVA (rmANOVA) for rejection rates revealed a marginally significant main effect of general rank-reversal $(F({1,54})=3.918,\; p= 0.053, \;{{\eta }_{p}}^{2}=0.068)$. When general rank-reversal was present, participants showed a higher tendency to reject the redistribution offer (${M}_{RR}= 0.238,\; {SD}_{RR}=0.022)$ compared to when it was absent $({M}_{NRR}= 0.221,\; {SD}_{NRR}=0.023)$.

With the Greenhouse–Geisser correction, a significant main effect of self-centered rank was observed on the rejection rate $(F(1.427,\; 77.037)=12.002,\; p <0.001 ,\; {{\eta }_{p}}^{2}=0.182)$. A post-hoc analysis with Bonferroni correction revealed higher rejection rates when participants’ self-centered rank decreased (${M}_{D}= 0.347,\; {SD}_{D}=0.043)$ compared to when it increased (${M}_{U}= 0.172,\; {SD}_{D}=0.025)$ or remained unchanged (${M}_{M}= 0.169,\; {SD}_{M}=0.026)$. The interaction effect of general rank-reversal and self-centered rank was also significant $(F({2,108})=6.293,\; p<0.001,\; {{\eta }_{p}}^{2}=0.104)$, showing higher rejection rates when general rank-reversal was present while self-centered rank remained constant.

**Figure 2:**
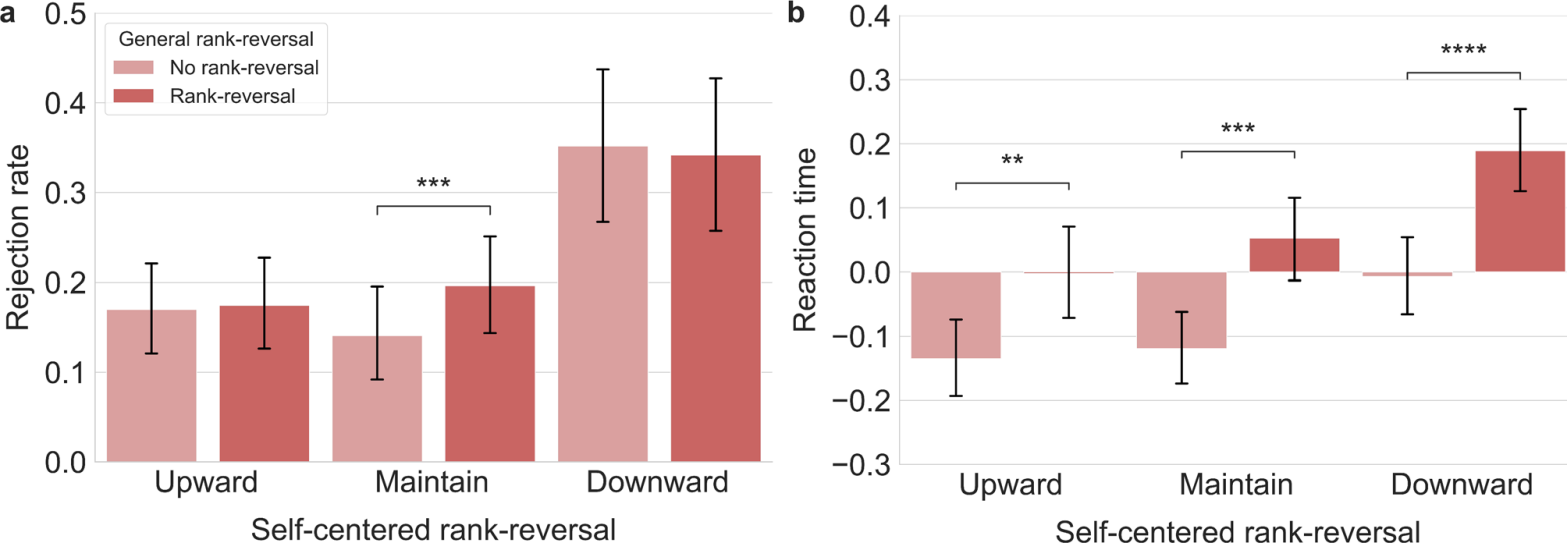
Group analysis on rejection rates and reaction time. (**a**) General rank-reversal increased rejection rates only when self-rank was maintained. (**b**) General rank-reversal delayed response time in all self-centered rank-reversal conditions. The error bars indicate the 95% confidence interval. **p < 0.01, ***p < 0.001, ****p < 0.0001.

To explore the effect of general rank-reversal among different self-centered ranks, a paired-sample *t*-test indicated that general rank-reversal aversion was expressed only when participants' self-centered rank remained constant throughout the redistribution process ($t(54)= 3.683,\; p=0.001,\; Cohe{n}{\text{'}}s d=0.497$) (Fig. 2a).

### General rank-reversal slows reaction time

For the reaction time, the 2 (General rank-reversal) $\times$ 3 (Self-centered rank-reversal) two-way rm ANOVA showed a significant main effect of general rank-reversal, $(F({1,51})=42.193,\; p<0.001,\; {{\eta }_{p}}^{2}=0.453)$ and a significant main effect of self-centered rank-reversal $(F({2,102})=7.302,\; p=0.001,\; {{\eta }_{p}}^{2}=0.125)$. Participants exhibited slower reaction times in general rank-reversal conditions (${M}_{RR}= 0.08,\; {SD}_{RR}=0.014)$, compared to no general rank-reversal conditions (${M}_{NRR}=-0.087,\; {SD}_{NRR}=0.013$). Post-hoc analysis with Bonferroni correction revealed slower RTs when participants’ self-centered rank decreased (${M}_{D}= 0.091,\; {SD}_{D}=0.027)$ compared to when it increased (${M}_{U}= -0.069,\; {SD}_{U}=0.026)$ or was maintained (${M}_{M}= -0.033,\; {SD}_{M}=0.024)$. No interaction effect was found between general and self-centered rank-reversal.

Furthermore, a paired-sample *t*-test indicated that general rank-reversal impacted RT for self-centered rank-reversal conditions. Results showed that for all self-centered rank-reversal conditions, RT became slower when general rank-reversal was present (${t}_{U}(51)= 3.168,\; p=0.003,\; Cohe{n}{\text{'}}s d=0.439;\; {t}_{M}(51)= 4.043,\; p<0.001,\; Cohe{n}{\text{'}}s d=0.561 ;\; {t}_{D}(51)= 5.658,\; p<0.001,\; Cohe{n}{\text{'}}s d=0.785)$ (Fig. 2b).

For a closer examination of reaction time, we analyzed the selection process by distinguishing between acceptance and rejection of the redistributed offer. A paired-sample *t*-test between general rank-reversal and no general rank-reversal condition revealed that only when participants accepted the redistributed offer, the presence of the general rank-reversal made the reaction time slower in all self-centered rank-reversal conditions (${t}_{U}(51)= 2.830,\; p<0.01,\; Cohe{n}{\text{'}}s d=0.392;\; {t}_{M}(51)= 3.794,\; p<0.001,\; Cohe{n}{\text{'}}s d=0.526 ;\; {t}_{D}(51)= 4.054,\; p<0.001,\; Cohe{n}{\text{'}}s d=0.562$).

### Individual differences in resource distribution behavior

After discovering that rank-reversal circumstances impact participants' redistribution behavior, we conducted a comparison of rejection tendencies among all individuals using binomial-GLMM. This included fixed effects of conditions (‘U’, ‘D’, interaction parameter of ‘G’ and ‘M’) as well as random effects of ‘U’, ‘D’, and the interaction parameter. The analysis showed significance for fixed effects of downward condition ($\beta =1.710,\; SE=0.362,\; p<0.001)$ and the interaction of general rank-reversal and maintain condition ($\beta =0.898,\; SE=0.255,\; p<0.001),$ and marginal significance for the upward condition ($\beta =0.657,\; SE=0.340,\; p=0.053)$. This suggests that the conditions for general and self-centered rank-reversal affected the choice of rejecting redistributed offers.

### Clustering individuals with diverse moral principles

The parameter estimates were utilized to explore variations in different redistribution motives among individuals. The k-means algorithm, a non-model-based method, was employed to define clusters. To estimate the optimal number of clusters k, the elbow method and silhouette score was used where both methods yielded the same result, k = 3 (average silhouette score 0.49). As a result, three clusters were yielded, where the most participants were assigned to Cluster 1 (${n}_{1 }=$ 28, silhouette score = 0.47), followed by Cluster 2 (${n}_{2}=$ 15, silhouette score = 0.28) and Cluster 3 (${n}_{3 }=$ 12, silhouette score = 0.82).

To gain deeper insights into each of the clusters, we conducted comprehensive analyses on both behavior and reaction time for individual clusters.

#### Cluster 1: Context-dependent rank-reversal aversion

**Figure 3:**
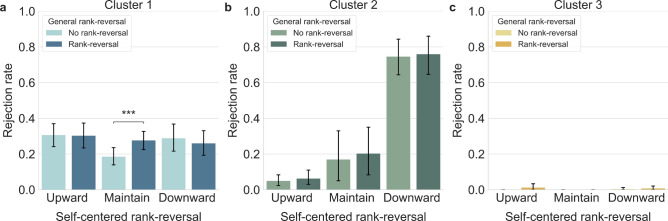
Clustered results on rejection rates. (**a**) Rejection rates of Cluster 1: General rank-reversal aversion was significant only in the maintain condition. (**b**) Rejection rates of Cluster 2: General rank-reversal aversion did not exhibit any difference in self-centered rank-reversal conditions. (**c**) Rejection rates of Cluster 3: Rejection rates for all conditions were close to 0. All error bars indicate the 95% confidence interval. ***p < 0.001.

In Cluster 1, a 2 (General rank-reversal) $\times$ 3 (Self-centered rank-reversal) two-way rm ANOVA on rejection rates revealed a significant interaction between general and self-centered rank-reversal $(F({2,54})=7.652,\; p= 0.001,\; {{\eta }_{p}}^{2}=0.221)$. A more detailed analysis through a non-parametric paired-sample *t*-test showed that the rejection rate increased significantly during general rank-reversal only when the self-rank was maintained ($Z=3.437,\; p<0.01,\; {M}_{NRRM}=0.186,\; {SD}_{NRRM}=0.136 ;\; {M}_{RRM}= 0.277,\; {SD}_{2}=0.142)$ (Fig. 3a).

**Figure 4:**
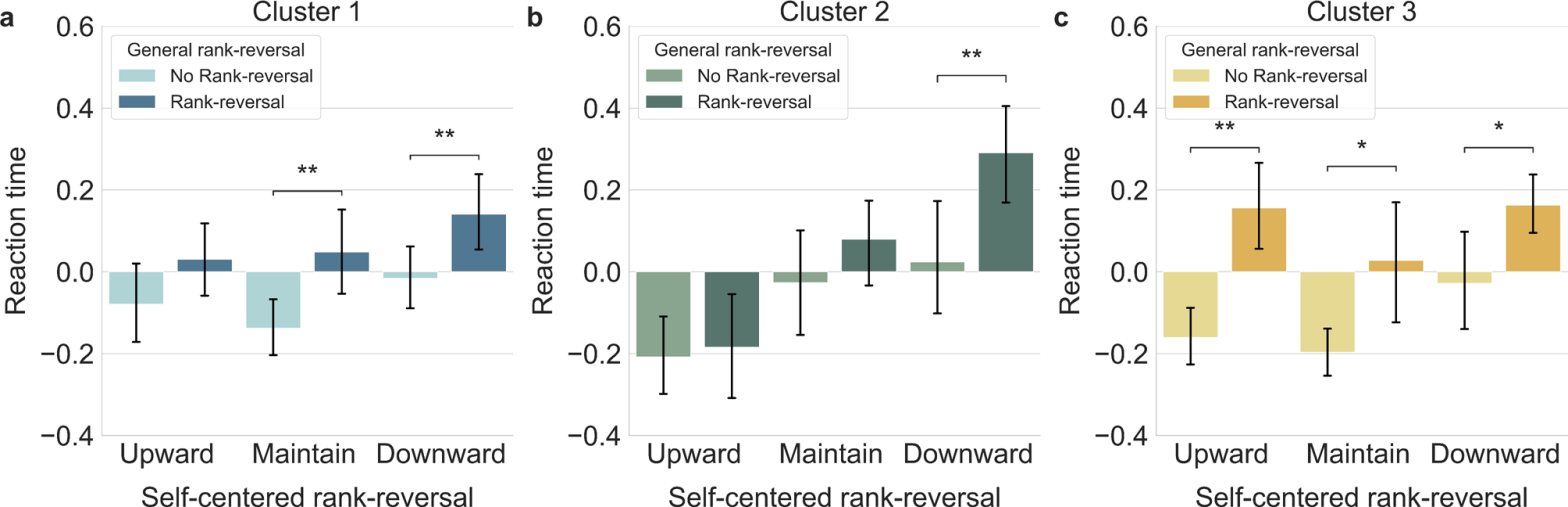
Clustered results on reaction time. (**a**) Reaction time of Cluster 1: General rank-reversal aversion slowed reaction time in the maintain and downward condition. (**b**) Reaction time of Cluster 2: General rank-reversal aversion slowed reaction time in the downward condition. (**c**) Reaction time of Cluster 3: All conditions showed delayed response due to general rank-reversal aversion. All error bars indicate the 95% confidence interval. *p < 0.05, **p < 0.01.

Examining reaction time in the same manner, the two-way rm ANOVA indicated only the main effect of general rank-reversal ($F({1,24})=16.817,\; p< 0.001,\; {{\eta }_{p}}^{2}=0.412).$ Further, a paired-sample *t*-test revealed the general rank-reversal significantly slowed down reaction time in the maintain and downward conditions, and almost significantly in the upward condition $({t}_{U}(24)= 1.890,\; p=0.071,\; Cohe{n}{\text{'}}s d=0.289;\; {t}_{M}(24)= 2.900,\; p=0.008,\; Cohe{n}{\text{'}}s d=0.321 ;\; {t}_{D}(24)= 3.573,\; p=0.002,\; Cohe{n}{\text{'}}s d=0.220)$ (Fig. 4a).

Participants in Cluster 1 displayed a higher rejection rate and slower reaction times when general rank was reversed, indicating a clear aversion to rank-reversal within this group.

#### Cluster 2: Prioritizing self-interest

In Cluster 2, a 2 (General rank-reversal) $\times$ 3 (Self-centered rank-reversal) two-way rm ANOVA on rejection rates showed a significant main effect only for self-centered rank ($F({2,28})=58.993,\; p< 0.001,\; {{\eta }_{p}}^{2}=0.808).$ Post-hoc analysis with Bonferroni correction revealed higher rejection rates for downward conditions (${M}_{D}= 0.753,\; {SD}_{D}=0.055)$, compared to upward (${M}_{U}= 0.057,\; {SD}_{U}=0.017)$ and maintain (${M}_{M}= 0.187,\; {SD}_{M}=0.072)$ conditions. However, the Wilcoxon test did not show any significant effect of general rank-reversal among self-centered rank conditions, indicating that general rank-reversal did not impact participants' behavior in any of the self-centered conditions (Fig. 3b).

For reaction time, a two-way rm ANOVA showed main effects for both general $(F({1,14})=13.533,\; p=0.002,\; {{\eta }_{p}}^{2}=0.492)$ and self-centered rank ($F({2,28})=7.712,\; p=0.002,\; {{\eta }_{p}}^{2}=0.355)$, with an almost significant interaction effect ($F({2,28})=2.776,\; p=0.079,\; {{\eta }_{p}}^{2}=0.165)$. Post-hoc analysis with Bonferroni correction showed faster RTs when participants’ self-centered rank increased (${M}_{U}= -0.196,\; {SD}_{U}=0.047)$ compared to when it decreased (${M}_{D}=0.157,\; {SD}_{D}=0.060)$ or was maintained (${M}_{M}= 0.026,\; {SD}_{M}=0.051)$. According to paired sample *t*-tests, general rank-reversal affected reaction time only in the downward condition (${t}_{D}(14)= 3.828,\; p=0.002,\; Cohe{n}{\text{'}}s d=0.269)$ (Fig. 4b).

Although Cluster 2 appeared to follow a specific decision-making process, prioritizing their own rank, the general rank-reversal did cause a delay in their reaction time, indicating the presence of general rank-reversal aversion, albeit not to a significant extent.

#### Cluster 3: Prioritizing inequality aversion

For participants in Cluster 3, rejection rates were close to zero, preventing a statistical analysis on these rates (Fig. 3c). However, the two-way rm ANOVA on reaction time revealed that general rank-reversal was a factor, with significant main effects for both general (${F}_{general}({1,11})=13.708,\; p=0.003,\; {{\eta }_{p}}^{2}=0.555)$ and self-centered rank $({F}_{self}({2,22})=4.187,\; p=0.029,\; {{\eta }_{p}}^{2}=0.276)$.

Furthermore, paired sample *t*-tests on all self-centered ranks demonstrated a significant effect of general rank conditions, indicating a slower reaction when general rank-reversal was present (${t}_{U}(11)= 3.867,\; p=0.003, \;Cohe{n}{\text{'}}s d=0.283;\; {t}_{M}(11)= 2.276,\; p=0.044,\; Cohe{n}{\text{'}}s d=0.342 ;\; {t}_{D}(11)= 2.302,\; p=0.042,\; Cohe{n}{\text{'}}s d=0.287)$ (Fig. 4c).

Despite the low rejection rate for the more equitable offer under all conditions, suggesting a strong aversion to inequality, the reaction time results indicate that general rank-reversal among choices is also considered by participants in this group.

#### Differences among clusters

To assess the validity of clustering, nonparametric tests were conducted on rejection rates in each condition. The Kruskal–Wallis test revealed significant differences in rejection rates for conditions involving general rank-reversal with upward (‘RRU’) or downward (‘RRD’) self-rank, and for conditions with no general rank-reversal with downward self-rank (‘NRRD’) (${H}_{RRU}(2,N=55)=36.874,\; p<0.001;\;{H}_{RRD}(2,N=55)=38.298,\; p<0.001;\;{H}_{NRRD}(2,N=55)=38.261,\; p<0.001)$.

For conditions with no general rank-reversal but with upward (‘NRRU’), maintained (‘NRRM’) self-rank, or with general rank-reversal and maintained self-rank (‘RRM’), only Cluster 1 and 2 were compared, excluding Cluster 3 with 0 rejection rate. The Mann–Whitney *U* test revealed significant difference in NRRU ($Mann{-}Whitney U=39,\; N=43,\; p<0.001)$ and RRM conditions ($Mann{-}Whitney U=118.5 ,\; N=43,\; p=0.019)$ but not under NRRM conditions, indicating that that Cluster 1 and 2 showed difference in rejection rates when ranks were not preserved.

A one-way ANOVA for normalized reaction times revealed significant differences among clusters only for the RRU condition ($F({2,49})=7.536,\; p=0.001,\; {{\eta }_{p}}^{2}=0.235)$. Post-hoc analysis using the Bonferroni correction showed that Cluster 2 (${M}_{2}= -0.184,\; {SD}_{2}=0.263)$ reacted faster than Cluster 1 and 3 (${M}_{1}= 0.030,\; {SD}_{1}=0.235;\;{M}_{3}= 0.156,\; {SD}_{3}=0.187 )$.

A one-way ANOVA examining potential age differences between clusters did not reveal any significant differences ($F({2,52})=0.703,\; p=0.5)$. A Chi-Square test to determine gender composition differences among the groups showed no significant association between gender and clusters (${\chi }^{2}(2,N=55)=1.569,\; p=0.456)$.

Significant differences in SVO were found among clusters ($F({2,52})=3.264,\; p=0.046,\; {{\eta }_{p}}^{2}=0.112)$. Post-hoc analysis using the Bonferroni correction indicated that Cluster 1 (${M}_{1}= 31.147, \;{SD}_{1}=10.623)$ exhibited higher SVO scores than Cluster 2 (${M}_{2}= 23.241, \;{SD}_{2}=7.095)$, suggesting a higher prosocial tendency.

## Discussion

In the pursuit of distributive justice, various conflicting motives, such as inequality aversion and rank-reversal aversion, have been proposed. However, previous research on rank-reversal aversion has primarily focused on third-party contexts, overlooking the impact on the self. To address this gap, our study investigated rank-reversal aversion in a first-party context. We found that rank-reversal aversion is evident only when one’s own rank is preserved but attenuated when it is not. Our results also highlight the diverse preferences individuals exhibit towards moral principles. While most participants chose to avoid rank-reversal when their own rank was maintained, some prioritized self-interest, and others advocated for absolute equality. Therefore, our study provides evidence that the expression of rank-reversal aversion can vary depending on the inclusion of self-rank, as well as the priorities of individuals.

Our study reinvestigated the rank-reversal aversion using a redistribution game introduced by Xie and colleagues^12^. The findings were successfully replicated but were limited to situations where the individual maintained their initial rank. Participants considered general rank-reversal, as indicated by increased reaction time regardless of their self-rank. When the self’s rank shifted downward, rejection of the redistributed offer increased despite the rank-reversal of others. The downward shift also led to a slowing of reaction time, suggesting conflicting motives. In upward conditions, both the pursuit of equality and self-interest led to the same decision—accepting the redistributed offer. In contrast, downward conditions required a choice between equality and self-interest, creating a conflict prompting value comparison and, consequently, increasing reaction time. Thus, along with behavioral tendencies, it appears that preserving one’s own rank had a higher priority over maintaining equality when self-rank decreased. Overall, these findings suggest that rank-reversal aversion does exist, though it may be outweighed by other motives such as self-interest.

We believe it is unlikely that a specific type of choice (rejection or acceptance) relies solely on automatic or deliberate decision-making processes. Rather, we assume that in certain situations, two or more conflicting values are simultaneously activated, and a higher-level valuation mechanism is engaged to consider additional external information to resolve the conflict between these values^17^. The additional operation of this higher-level valuation mechanism could be the primary cause of increased reaction times. Based on this theoretical background, the reaction time results of this study indicate that in the rank-reversal condition compared to no rank-reversal condition, the value of avoiding rank-reversal is additionally activated. This conflicts with other values (the pursuit of self-interest and the avoidance of inequality), resulting in longer reaction times regardless of the type of choice across all groups. Additionally, participants are generally more strongly motivated to accept resource redistribution proposals to resolve unfairness, so the reaction times are faster when the choice to elevate their own status (upward conditions) aligns with the acceptance behavior. Conversely, reaction times are slower when the choice to avoid lowering their status (downward conditions) conflicts with acceptance behavior.

Taking our investigation a step further, we examined individual variations in behavior and uncovered distinct moral principles held by people, recognizing that people may espouse diverse beliefs about moral decision-making^18,19^. The first cluster, comprising the majority of participants, displayed both rank-reversal aversion and inequality aversion. They exhibited increased rejection rates in general rank-reversal conditions when their own rank was preserved, while showing inequality aversion in self-centered rank-reversal conditions. A low rejection rate was anticipated in upward conditions, as accepting the redistributed offer could align with both self-interest and equality. However, Cluster 1 showed little disparity in rejection rates between upward and downward conditions. This unaltered rejection rate in upward conditions can be attributed to advantageous inequality aversion, which involves aversion to situations where one receives more than others^1,3^. Previous research have shown that individuals in advantaged positions are willing to relinquish their benefits to reduce inequality and promote a more equitable environment^3,20^. This aversion to advantageous inequality may have contributed to relatively high rejection rates in upward conditions for Cluster 1. Moreover, the reaction time results indicate that individuals recognized the rank-reversal of others, especially when their own rank was maintained or decreased. The hesitation observed in the downward condition may reflect the dilemma between selfish and prosocial motives, as rejecting the offer could satisfy one's selfish desires but fail to uphold principles of equality. Consequently, Cluster 1 exhibited a combination of attitudes towards both rank-reversal aversion and inequality aversion, resulting in a relatively consistent rate of rejection across conditions.

The second cluster demonstrated a pronounced inclination toward self-interest. They rejected the redistributed offer if their self-rank decreased, irrespective of the rank-reversal experienced by others. Despite the decisions having no impact on their earnings, they resisted being in last place while being willing to accept to be in first or second place. This behavior can be attributed to last-place aversion, wherein individuals avoid being in the last position even at the expense of equality^16^. Survey results corroborated their behavior, revealing a low prosocial tendency^21^. Nevertheless, general rank-reversal was not entirely ignored in this group, as evidenced by the slowing down of reaction time when the general rank-reversal was present. Overall, while Cluster 2 acknowledged general rank-reversal, it did not exert enough influence to alter their preference for self-interest.

The third cluster also showed a robust decision rule, emphasizing a significant commitment to achieving equality. Unlike Cluster 1, who adhere to the equality norm but factored in self-rank when assessing it, Cluster 3’s pursuit of equality was not tied to one's status but rather to the absolute difference in points between participants. In all circumstances, rejection rates converged to zero, indicating that the majority of redistributed offers were accepted. Reaction time analysis revealed that general rank-reversal was considered, as the presence of rank-reversal slowed decision-making for all self-centered ranks. In summary, Cluster 3 comprised individuals who pursued the equality norm but focused on reducing the earnings gap without centering it on the self.

SVO questionnaires assess the extent to which individuals exhibit concern for others, with the scale ranging from “prosocial” to “competitive”^21^. It presupposes a relatively straightforward scenario involving the distribution of resources between oneself and the other, where the competing motivations are limited to self-interest and prosocial distributions. However, the experiment we have conducted introduces an additional prosocial motivation where participants also need consider whether to accept or reject the status reversal between the other participants. Therefore, if a higher SVO score reflects the ability to learn and adapt to a variety of social norms, it is reasonable to conclude that Cluster 1 exhibited the highest SVO scores, given their consideration of multiple prosocial motives. In contrast, Cluster 3 adhered to a single standard, inequality aversion. Additionally, individuals with high SVO scores would allow others to receive more resources than themselves, a choice that is unlikely to be preferred by individuals with extreme inequality aversion, as seen in Cluster 3.

The current findings suggested that rank-reversal aversion becomes evident only when the rank of the self is unaffected. Furthermore, individuals exhibit distinct moral principles, with some showing high selfish motives (Cluster 2) or displaying a strong preference for equality (Cluster 3), while others manifest a combination of rank-reversal aversion and inequality aversion with a touch of self-interest (Cluster 1). Although the presence of rank-reversal was acknowledged, the ways in which it influenced behavior varied. These findings provide new insight that the self plays a significant role in the manifestation of rank-reversal aversion, as it may diminish when the self-rank changes. Additionally, this study underscores the idea that examining social decisions as a group may not be suitable, given the diversity of individual moral principles.

This study as several limitations. First, questions may arise regarding why all participants started at second place. In reality, individuals span multiple income levels, ranging from rich to poor. However, our study aimed to investigate the impact of rank transfer within one individual and to evaluate whether upward or downward rank movement would alter their behavior. Therefore, participants were assigned to the middle in order to assess the study's primary objectives. Future research could explore whether the difference of initial rank affects the manifestation of rank-reversal aversion. Secondly, the use of deception may raise concerns about the potential for contamination of participants in future studies. This approach was intended to circumvent any suspicion on the part of the participants with regard to future studies conducted in restricted environments, such as using fMRI, while allowing participants vividly perceive that they were interacting with real people online and could influence their monetary gains. Future studies should aim to replicate the present findings in a more natural environment without the use of deception. Lastly, previous studies have shown that revealing names of others can increase the amount of distribution in dictator games while not affecting the payoffs in ultimatum games^22^. This suggests that disclosing identities influences intuitive prosocial motivations but not strategic decision making. Similarly, our experiment resembles a dictator game where participants’ choices could not be altered by other players. Therefore, revealing the names of other two participants could have activated intuitive prosocial motivations, particularly affecting choices involving self-interest. To determine if names influence participants’ choices in our scenario, future research should replicate the experiment without revealing any names.

The current study can be extended into political, economic, and social domains. Opposition to redistribution policies often stems from a preference to maintain the status quo^10^. For instance, individuals earning just above the minimum wage may resist an increase in minimum wage, fearing a loss of their status above last-place^16^. Furthermore, individuals who faced the risk of rank-reversal expressed greater opposition to redistribution polices compared to those who were not at risk^23^. The current study suggests that this opposition may not solely stem from the fear of losing rank. In Cluster 1, participants exhibited a similar level of opposition to moving upwards and downwards, indicating an attempt to maintain equality by refusing unfair benefits. Similarly, individuals tend to support policies that reduce their own privilege, refusing advantageous inequality, rather than reducing the disadvantage of others^20^. Therefore, decision-making related to redistribution appears to depend on more than just self-interest or equality but also involves a more complex calculation. Understanding the mechanisms underlying redistribution can contribute to the development of more effective policies that meet the needs of individuals at all levels.

In conclusion, this study demonstrated the existence of rank-reversal aversion in a first-party context, highlighting the impact of self-intervention on redistribution decisions. Our findings suggest that individuals may vary in their application of moral principles, offering a more nuanced insight into the understanding of distributive justice.

## Methods

### Participants

Sixty-one participants (24 male; mean age = 22.9, ranging from 19 to 31 years) took part in the study, recruited through the Korea University student community website. Screening ensured no history of prior enrolment in advanced psychology courses. The study and subsequent follow-up survey were conducted online using the JATOS application^24^ and Qualtrics software (version August, 2023), respectively. Six participants were excluded from the analysis: three misunderstood instructions, two failed the catch trial test, and one was removed as an outlier (IQR = − 0.247, 0.695). This left 55 participants (20 male, mean age = 22.7, ranging from 19 to 31 years) for analysis. In addition, for reaction time analysis, three participants were excluded as outliers (IQR = − 1.603, 6.496) after standardizing reaction time.

A priori power analysis, conducted with G*Power 3.1.9.7^25^, determined the appropriate sample size. The effect size (${{\eta }_{p}}^{2}$= 0.064) was obtained from an independent pilot study conducted prior to the main experiment (see Supplementary Sect. 1). The power analysis for a repeated-measures ANOVA testing for within-subject factors indicated that a sample size of N = 26 at α = 0.05 with 95% power was sufficient to detect a medium effect^25^. The study protocol was approved by the Institutional Review Board of Korea University and was conducted in accordance with the Declaration of Helsinki. All participants provided written informed consent before the experiment, and upon completion, they received 10,000 KRW (= 7.46 USD) and underwent a comprehensive debriefing process.

### Task design and procedure

The task was derived from the redistribution game originally developed by Xie and colleagues^12^. In this game, participants assumed the role of third-party decision-makers tasked with deciding whether to redistribute a fixed sum of money from the rich to the poor. Some conditions involved a hierarchy reversal, where the redistribution choice reversed the income distribution between two individuals.

In the modified version of the redistribution game, the participants’ earnings were integrated, transforming the original task into a first-party context. The players chose either to redistribute or refrain from doing so while maintaining their original earnings. Consequently, the redistributed offer could either involve the reversed hierarchy of others (general rank-reversal condition) or not (no general rank-reversal condition). Simultaneously, it could include the reversed hierarchy of self (self-centered rank-reversal: upward, downward) or not (self-centered rank-reversal: maintain), resulting in six conditions.

Each trial commenced with a brief fixation point onset, followed by the presentation of the initial offer (e.g., A: 10 points, B: 4 points, You: 5 points) and the redistributed offer (e.g., A: 6 points, B: 8 points, You: 5 points), each displayed for two seconds. Subsequently, participants made their choice between the two payments by pressing the ‘a’ key for the initial payment or the ‘l’ key for the redistributed payment (Fig. 1).

Participants were informed that they were engaging in a game with two other players, each independently participating as a distributer. After all three players completed the game, their choices would be randomly selected and applied to their final payments. For instance, participants enter the game believing two others are online simultaneously. The instructions explain that each participant independently engages in the redistribution game, choosing between two computer-generated offers. After all three participants complete the game, a random selection of choices determines the final payments. In reality, the other participants are simulated. We anticipate this setup will stimulate various social motivations, as each participant assumes their choices may affect both themselves and others, especially given their belief that real people are playing alongside them. To enhance the credibility of the manipulation, participants were shown the names of other players before starting the game; however, there were no actual other players, and every participant ultimately received the same amount of money.

Additionally, catch trials were designed to differentiate between participants genuinely seeking equality and those exhibiting insincere behavior. In these catch trials, the redistributed payment deviated from other trials, making the initial payment more equal. Therefore, participants who truly committed to equality would need to select the initial payment. Those who consistently pressed the same key (indicating the redistributed payment) even during catch trials were categorized as insincere participants and was subsequently excluded from the analysis. Following the identification and exclusion of insincere participants using catch trials, the trials themselves were omitted from the analysis.

To assess participants’ psychological traits, self-report questionnaires such as the social value orientation scale^21^, social dominance orientation scale^26^, social desirability scale^27^, and the social comparison scale^28^ were administered through the online survey tool Qualtrics software (version August, 2023), following their completion of the modified redistribution game. Participants were not provided additional compensation for completing the SVO survey, and they were instructed to imagine hypothetical scenarios during the process. Only significant results are reported.

### Data acquisition and statistical analysis

All statistical analyses were conducted using R software (version 4.2.2^29^, R Studio^30^) and SPSS 27.0 (Statistical Package for the Social Sciences, IBM Corp., Armonk, NY, USA). Data visualization was performed via Python, using the Matplotlib^31^ and Seaborn^32^ package.

The significance level was set at α = 0.05 (two-tailed), and the Greenhouse–Geisser correction was applied when the sphericity assumption was violated. Non-normal data were analyzed using the Kruskal–Wallis test, the Mann–Whitney *U* test, and the Wilcoxon rank-sum test. When needed, Cohen’s *d* and partial eta-squared were reported as effect size measures.

To explore the impact of conditions on decisions in the main task, a 2 (General rank-reversal: Yes or No) $\times$ 3 (Self-centered rank-reversal: upward, maintain, downward) two-way repeated-measures analysis of variance (rm ANOVA) was conducted on rejection rates and reaction times. Reaction time data were standardized within each participant across all trials. To assess rank-reversal aversion, paired-sample *t*-tests were performed between general rank-reversal and no general rank reversal conditions for each self-centered rank-reversal condition.

Individual difference for distribution choice was examined by a General Linear Mixed Model (GLMM), with the ‘glmer’ function in the ‘lme4’ package^33^. The GLMM tested whether conditions influenced the binary response to the redistributed offer (1: reject, 0: accept). Model selection was based on the Akaike Information Criterion (AIC), where the model with a lower AIC indicates better support from the data^34^. The final model included fixed effects of upward (U), downward (D), and the interaction of maintain (M) and general rank-reversal (G) conditions as categorical variables, where they were coded as 1 if each condition were present and 0 if they were not. Random effects also included the upward, downward, and interaction parameter of maintain and general rank reversal conditions.

K-means cluster analysis was employed to classify motivations for redistributions based on experimental data^35^. The elbow method and silhouette score determined the optimal number of clusters, assessed using the ‘cluster’ package^36^ and ‘factoextra’ package^37^ in R. Cluster validity was confirmed through nonparametric tests on rejection rates and one-way ANOVA on reaction time for each condition.

Pearson correlation analysis examined the relationship between self-report measures of personal traits and GLMM coefficient estimates. Additionally, a one-way ANOVA was conducted using the clusters as factors to explore potential differences in personal traits among clusters.

### Supplementary Information

Supplementary Information.

## Data Availability

The datasets generated during and/or analyzed during the current study are available in the OSF repository, https://osf.io/5f47m/?view_only=fda21c0baaa840269d0d489bd1fa3157.
