# Supplementary material for: Self-interest overrides rank-reversal aversion in resource distribution
Source: Sci Rep. 2024 Aug 24;14:19704. doi: 10.1038/s41598-024-70225-9 (PMC11344805; doi:10.1038/s41598-024-70225-9)
Supplement: Supplementary file 1 — Supplementary Information. [file 41598_2024_70225_MOESM1_ESM.pdf]

SUPPLEMENTARY INFORMATION

Self-Interest Overrides Rank-Reversal Aversion in Resource Distribution

Minyoung Kim[1], Kun Il Kim[1], Hackjin Kim[1*]

[1]Korea University, School of Psychology, Seoul, 02841, Republic of Korea.

[*]**Correspondence**

Hackjin Kim, Ph.D.

School of Psychology

Korea University

145 Anam-ro, Seongbuk-gu, Seoul 136-701, Republic of Korea

hackjinkim@korea.ac.kr

Section 1: Pilot study

Participants An independent pilot study was conducted to assure replication of the study and determine the effect size for the main experiment. Nineteen participants took part in the study, where three were excluded from the analysis due to the risk of insincere attitudes. Since it was difficult to discriminate insincere participants to equal seeking ones, we excluded all participants that showed consistent behavior of choosing the same choices repeatedly. This later led to the implementation of catch trial tests in the main experiment.

Materials and Procedure The task was identical to the main task, only with the absence of catch trials.

Results The 2 (General rank-reversal) × 3 (Self-centered rank-reversal) two-way rm ANOVA for rejection rates revealed a significant main effect of self-centered rank-reversal $(F(2,30) = 7.809, p = .002, {\eta_p}^2 = 0.342)$ but no interaction or main effect of general rank-reversal was found. To gain a more detailed understanding of individual data patterns, we employed a binomial-GLMM (1 for rejecting redistribution offer, 0 for accepting) with information of conditions as categorical variables ('G', 'U', 'D') for both fixed and random effects (Supplementary Table S1). Subsequently, utilizing the parameter estimations, we applied a k-means clustering algorithm, resulting in identifying two clusters. Cluster 1, consisting of 9 participants exhibited rank-reversal aversion to general ranks depending on self-centered rank conditions (Supplementary Fig. S1a; Supplementary Table S2). Cluster 2, comprising 7 participants, prioritized self-interest with increased rejection rates as self-rank went downward. (Supplementary Fig. S1b; Supplementary Table S3). Although further statistical significance among the conditions could not be established due to the small sample size and limited data, we could infer that individuals apply different moral principles when allocating resources.

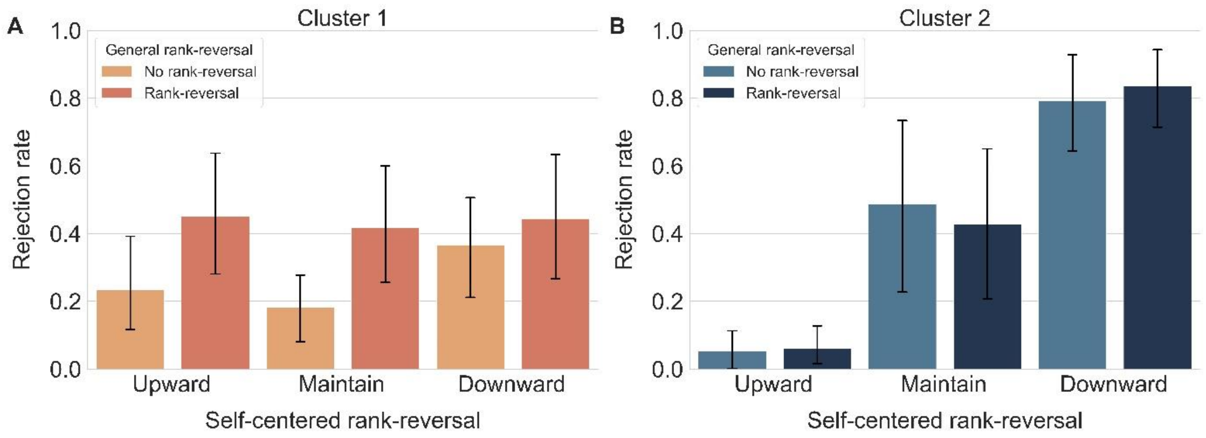

Supplementary Fig.S1 Rejection rates of each cluster for the pilot study. (a) Cluster 1 showed increased rejection rates for general rank-reversal condition. (b) Cluster 2 showed increased rejection rates according to self-rank.

Fixed effects	**Effect**	**b(SE)**
	Intercept	-0.9735(0.3767) **
	General rank-reversal	0.5940(0.3756)
	Upward	-1.2315(0.4912) *
	Downward	1.1538(0.4976) *
Random effects	**Effect**	**Variance (SD)**
	Intercept	2.018(1.420)
	General rank-reversal	1.969(1.403)
	Upward	3.181(1.784)
	Downward	3.596(1.896)
Model Statistics	AIC	1929.0
	BIC	2006.8
	Loglikelihood	-950.5

Supplementary Table S1 General linear mixed model for the pilot study. Model description was as follows: Choice (1: reject, 0: accept) ~ General rank-reversal (1: present, 0: absent) + Upward (1: present, 0: absent) + Downward (1:present, 0:absent) + (General rank-reversal + Upward + Downward | subject). AIC and BIC indicate the Akaike Information Criterion and Bayesian Information Criterion, respectively. *$p < 0.05$, **$p < 0.01$

Source	df_{Num}	df_{Den}	SS_{Num}	SS_{Den}	F	p	η_p^2
General rank-reversal	1	8	0.420	1.826	1.839	.212	.187
Self-centered rank-reversal	1.151	9.204	0.103	0.837	0.985	0.360	.110
General rank-reversal x Self-centered rank-reversal	2	16	0.066	0.104	5.080	0.020*	.388

Supplementary Table S2 Repeated measures ANOVA results for Cluster 1. df_{Num} and df_{Den} indicates degrees of freedom numerator and degrees of freedom denominator, respectively. SS_{Num} and SS_{Den} indicates sum of squares numerator and sum of squares denominator, respectively. *$p < 0.05$

Source	df_{Num}	df_{Den}	SS_{Num}	SS_{Den}	F	p	$\eta_p{}^2$
General rank-reversal	1	6	0.000	0.029	0.015	.905	.003
Self-centered rank-reversal	2	12	4.026	1.403	17.219	.000**	.742
General rank-reversal x Self-centered rank-reversal	2	12	0.019	0.067	1.655	.232	.216

Supplementary Table S3 Repeated measures ANOVA results for Cluster 2. df_{Num} and df_{Den} indicates degrees of freedom numerator and degrees of freedom denominator, respectively. SS_{Num} and SS_{Den} indicates sum of squares numerator and sum of squares denominator, respectively. $^{**}p < 0.01$
